# RNA-Seq of Huntington’s disease patient myeloid cells reveals innate transcriptional dysregulation associated with proinflammatory pathway activation

**DOI:** 10.1093/hmg/ddw142

**Published:** 2016-05-11

**Authors:** James R. C. Miller, Kitty K. Lo, Ralph Andre, Davina J. Hensman Moss, Ulrike Träger, Timothy C. Stone, Lesley Jones, Peter Holmans, Vincent Plagnol, Sarah J. Tabrizi

**Affiliations:** 1 Department of Neurodegenerative Disease, UCL Institute of Neurology, University College London, London, WC1N 3BG, UK; 2 UCL Genetics Institute, University College London, London, WC1E 6BT, UK; 3 MRC Centre for Neuropsychiatric Genetics and Genomics, School of Medicine, Cardiff University, Cardiff, CF24 4HQ, UK

## Abstract

Innate immune activation beyond the central nervous system is emerging as a vital component of the pathogenesis of neurodegeneration. Huntington’s disease (HD) is a fatal neurodegenerative disorder caused by a CAG repeat expansion in the huntingtin gene. The systemic innate immune system is thought to act as a modifier of disease progression; however, the molecular mechanisms remain only partially understood. Here we use RNA-sequencing to perform whole transcriptome analysis of primary monocytes from thirty manifest HD patients and thirty-three control subjects, cultured with and without a proinflammatory stimulus. In contrast with previous studies that have required stimulation to elicit phenotypic abnormalities, we demonstrate significant transcriptional differences in HD monocytes in their basal, unstimulated state. This includes previously undetected increased resting expression of genes encoding numerous proinflammatory cytokines, such as *IL6*. Further pathway analysis revealed widespread resting enrichment of proinflammatory functional gene sets, while upstream regulator analysis coupled with Western blotting suggests that abnormal basal activation of the NFĸB pathway plays a key role in mediating these transcriptional changes. That HD myeloid cells have a proinflammatory phenotype in the absence of stimulation is consistent with a priming effect of mutant huntingtin, whereby basal dysfunction leads to an exaggerated inflammatory response once a stimulus is encountered. These data advance our understanding of mutant huntingtin pathogenesis, establish resting myeloid cells as a key source of HD immune dysfunction, and further demonstrate the importance of systemic immunity in the potential treatment of HD and the wider study of neurodegeneration.

## Introduction

Huntington’s disease (HD) is an incurable, autosomal dominant neurodegenerative disorder that is caused by a CAG repeat expansion in exon 1 of the huntingtin (*HTT*) gene (1). An expansion of 40 or more repeats is fully penetrant, resulting in production of the mutant (m)HTT protein that is the primary cause of cellular death and dysfunction (2). The central nervous system has traditionally been regarded as the primary site of HD pathology, with patients suffering from a range of symptoms including movement disorders, cognitive impairment and psychiatric disturbance. However, it is apparent that HD is in fact a disease of the whole body, with the mHTT protein being expressed in all cells and tissues that have been studied (3). Patients are affected by a range of peripheral pathologies including skeletal muscle wasting (4), and there is mounting interest in the immune system as a potential modifier of disease progression.

HD patients exhibit immune dysfunction both centrally, in the form of microglial activation (5), and peripherally, where elevated levels of proinflammatory mediators are detectable up to 16 years before the predicted onset of motor symptoms (6,7). These phenomena have recently been found to be correlated, suggesting a global immune response to the presence of mHTT whereby central pathology is mirrored peripherally (8). The innate immune system is thought to be the primary source of HD immune dysfunction, with myeloid cells comprising circulating monocytes and tissue macrophages the likely peripheral effector cells (9). HD myeloid cells *ex vivo* are hyper-reactive, producing significantly more IL-8 and TNFα following lipopolysaccharide (LPS) stimulation (10), and also exhibit functional deficits in their migratory and phagocytic capabilities (11,12). These changes contrast with T lymphocytes of the adaptive immune system, which do not display any intrinsic phenotypic defects as a result of mHTT expression (13). Such alterations in the innate immune system are mirrored in mouse models of HD (12), suggesting that they are caused by a common pathological effect of mHTT expression. Indeed, several studies have demonstrated that mHTT has a cell-autonomous effect on innate immune cell function, and that immune activation in HD is not simply a secondary response to neuronal pathology (10,14). For example, the hyper-reactive phenotype of human HD myeloid cells is reversible following HTT lowering with siRNA, while mHTT levels in peripheral immune cells correlate with disease burden scores in HD patients (15). Importantly, the peripheral innate immune system has been suggested to act as a modifier of HD progression. Dampening of the immune system using a peripherally restricted CB2 agonist improves the phenotype of an HD mouse model (16), as does the administration of a TNFα antagonist (17), a KMO inhibitor (18) and bone marrow transplantation (19). Although these data suggest that the study of the HD peripheral immune system has disease relevance far beyond its uses as a ‘window’ into the brain, the mechanisms underlying HD immune dysfunction remain incompletely understood.

To date, studies on peripheral HD myeloid cells have almost exclusively demonstrated phenotypic abnormalities in response to LPS stimulation (10,11), and there is very little information available on whether these cells are also abnormal in their basal, unstimulated state. Indeed, there is currently no evidence that HD myeloid cells have increased resting expression of proinflammatory cytokines (10,14). It is therefore unclear whether mHTT only affects HD myeloid cells following stimulation, or if there is an underlying mechanism producing resting dysfunction that is responsible for the phenotypic changes seen once the cells are activated. Mutant HTT has been found to have a cell-autonomous priming effect on HD murine microglial cell lines (14), resulting in increased proinflammatory gene expression in the absence of stimulation. Although a similar effect is yet to be shown in primary human cells, it raises the possibility that mHTT’s potential baseline effects in HD myeloid cells are responsible for their hyper-reactive response to stimulation. This has important implications for targeting peripheral immunity as a therapeutic for HD, as intrinsic pathology is more likely to be treatable without the need to time intervention to coincide with inflammatory events.

Regardless of whether mHTT’s pathogenic effects are innate or only occur following stimulation, evidence will likely be detectable as changes in the cellular transcriptome; if mHTT has a tonic pathogenic effect then transcriptional changes should occur in unstimulated, resting cells. Transcriptional dysregulation is a central feature of HD pathogenesis, and has been consistently demonstrated in a range of HD tissues (20,21). Such broad effects on transcription could be caused by several underlying causative mechanisms, including sequestration of transcription factors by mHTT aggregates, impairment of transcription factor degradation by the ubiquitin-proteasome system, changes in histone modifications and the direct binding of mHTT to DNA (22). Moreover, the functions of upstream intracellular signalling pathways are likely affected. Mutant HTT activates the NFκB signalling pathway via a direct interaction with the IKK complex, a key regulator of the NFκB family of transcription factors (23). This is associated with hyper-reactivity in HD myeloid cells, which exhibit an exaggerated and prolonged NFκB signalling response to LPS (10). However, it is not clear whether HD myeloid cells also display increased NFĸB activity in the resting state; this is likely key to determining whether HD myeloid cells are also abnormal in the absence of stimulation. Furthermore, signalling downstream of the LPS receptor TLR4 is complex, including as it does activation of signalling pathways such as those involving the mitogen-activated protein kinases (MAPKs) (24), the contribution of which to HD myeloid cell dysfunction is yet to be fully explored.

Previous gene expression studies have demonstrated numerous transcriptional changes in HD peripheral blood cells (21,25,26), but these have been largely carried out on heterogeneous cell populations with varying transcriptional profiles, and have rarely been validated between studies. Therefore, study of specific cell populations using modern sequencing technology is required to identify biologically relevant transcriptional changes associated with mHTT expression. Here, we use RNA sequencing (RNA-Seq) to investigate the human HD monocyte transcriptome under both resting and LPS-stimulated conditions. We demonstrate that the transcriptome of HD myeloid cells is abnormal even in the absence of stimulation, including previously unreported increases in the basal expression of proinflammatory cytokines. Pathway analysis to dissect the biological relevance of these expression changes identifies numerous proinflammatory functional gene sets that are altered in unstimulated HD myeloid cells, as well as specific upstream regulator molecules that may be driving transcriptional change; activation of potential regulator molecules is further investigated using Western blotting. These data provide new insight into the pathogenesis of mHTT expression in systemic innate immune cells, in addition to the potential for modulating the peripheral immune system to modify disease progression.

## Results

### HD monocytes exhibit resting proinflammatory transcriptional changes

Although the phenotypic abnormalities associated with HD myeloid cells are relatively well characterized, considerably less is known about the transcriptional profile that underpins these changes. To address this, CD14^+ ^monocytes were isolated from peripheral blood samples donated by thirty manifest HD patients and thirty-three control subjects, and cultured with and without stimulation with LPS and interferon gamma (IFN-γ). RNA-Seq of these samples was carried out to provide a quantitative analysis of the entire HD monocyte transcriptome. LPS-stimulation was confirmed by pooling the HD and control samples and performing differential expression analysis between the unstimulated and stimulated datasets; 12 599 genes were found to be differentially expressed (false discovery rate (FDR) < 0.05; Dataset S1).

**Figure 1.**
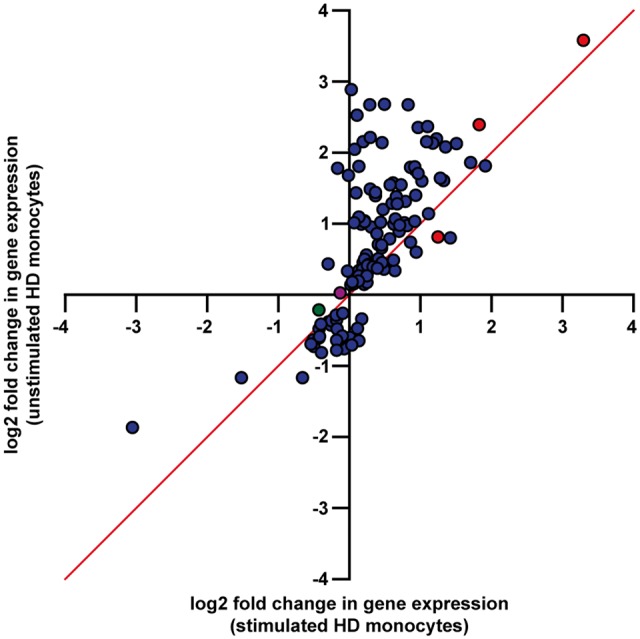
Comparison of log2 fold changes in resting and stimulated HD and control monocytes. The log2 fold changes for the 130 differentially expressed genes in resting HD monocytes (FDR < 0.05) were compared between the unstimulated and stimulated datasets. The magnitude of the relative expression difference was greater in the unstimulated samples for 116 of these genes, suggesting that transcriptional differences between HD and control monocytes become less pronounced in response to stimulation. Genes which were only differentially expressed in the unstimulated samples are shown in blue, while the three genes which were differentially expressed in both datasets are shown in red. *HTT* is further shown in green, while the NFĸB transcription factor *RELA* (p65) is shown in purple (however neither of these genes were differentially expressed). The line x = y is also shown.

Differential expression analyses were then performed to determine which genes are significantly altered between HD and control monocytes, whereby the unstimulated and stimulated monocytes were analysed separately. Analysis of the unstimulated monocyte data identified 130 genes that were differentially expressed (FDR < 0.05, 101 upregulated and 29 downregulated) in resting HD cells compared with control (Table 1; Dataset S2). Although the differentially expressed genes were associated with a wide range of effector functions, proinflammatory cytokines and chemokines were heavily featured, with HD monocytes having significantly increased expression of *IL6*, *IL12B*, *IL19*, *IL23A*, *CCL8*, *CCL19*, *CCL20*, *CXCL6* and *CSF2* gene transcripts. All of these genes had a log2 fold change of > 1, corresponding to a >2-fold increase in mRNA expression. This contrasted with the stimulated dataset, which showed comparatively little evidence of differential expression between HD and control cells, as only *DNAJB13*, *STAC* and *RASEF* were found to be differentially expressed (FDR < 0.05; Dataset S3). Each of these genes were also found to be differentially expressed in the unstimulated dataset (these genes are displayed as red dots in Fig. 1). Comparison of the log2 fold changes revealed a general trend whereby the relative expression differences between HD and control were lower in the stimulated compared with the unstimulated dataset; this was the case for 116 of the 130 differentially expressed genes (Fig. 1). This observation of more profound change in resting HD monocytes is in marked contrast to previous functional studies where a stimulus has been necessary to elicit phenotypic differences (10–12).

**Table 1.** The top 20 gene expression changes in resting HD monocytes (ranked by FDR)

Gene name	Ensembl ID	RPKM (control)	RPKM (HD)	Log2 fold change	*P*-value	FDR	Protein function
*FAM124A*	ENSG00000150510	0.242	0.812	2.684	4.61E-08	6.05E-04	Functionally uncharacterized
*IL19*	ENSG00000142224	0.586	1.473	2.358	1.15E-07	7.57E-04	Proinflammatory cytokine
*IL23A*	ENSG00000110944	1.914	3.597	1.576	7.84E-07	2.55E-03	Proinflammatory cytokine
*FAM213B*	ENSG00000157870	7.837	5.201	−0.604	8.99E-07	2.55E-03	Prostaglandin metabolism
*TGFA*	ENSG00000163235	0.665	1.392	1.550	9.69E-07	2.55E-03	Growth factor; promotes cell proliferation
*FZD7*	ENSG00000155760	0.397	0.246	−0.721	1.39E-06	3.04E-03	Wnt protein receptor
*C6orf223*	ENSG00000181577	0.063	0.297	2.127	1.92E-06	3.60E-03	Functionally uncharacterized
*PROCR*	ENSG00000101000	3.695	10.872	1.552	3.61E-06	5.69E-03	Receptor for activated protein C
*NT5E*	ENSG00000135318	0.210	0.725	2.082	4.24E-06	5.69E-03	Hydrolyses extracellular nucleotides
*SMO*	ENSG00000128602	0.259	0.124	−1.161	4.33E-06	5.69E-03	G protein-coupled receptor
*CISH*	ENSG00000114737	2.723	5.161	1.439	5.90E-06	7.04E-03	Negative regulation of JAK/STAT signalling
*C6orf165*	ENSG00000213204	0.038	0.086	1.205	6.57E-06	7.20E-03	Functionally uncharacterized
*CCL19*	ENSG00000172724	0.518	1.767	1.763	8.79E-06	8.89E-03	Chemoattraction of T and B lymphocytes
*PTGS2*	ENSG00000073756	2.319	18.604	1.813	9.52E-06	8.93E-03	Prostaglandin synthesis enzyme
*HPSE*	ENSG00000173083	11.322	24.941	1.142	1.10E-05	9.68E-03	Extracellular matrix remodelling
*VEGFA*	ENSG00000112715	5.475	14.251	0.898	1.35E-05	1.11E-02	Promotes angiogenesis and endothelial cell growth
*ANXA11*	ENSG00000122359	136.875	105.621	−0.374	1.72E-05	1.33E-02	Phospholipid-binding protein
*CDK2*	ENSG00000123374	5.534	7.186	0.390	1.88E-05	1.39E-02	Cell cycle regulation
*R3HCC1*	ENSG00000104679	15.225	11.394	−0.421	2.18E-05	1.45E-02	Nucleic acid binding
*PGAP3*	ENSG00000161395	2.943	2.310	−0.364	2.28E-05	1.45E-02	GPI-specific phospholipase

### HD monocytes do not display significant changes in alternative splicing

Next, we investigated whether changes in alternative splicing underlie the expression differences seen in our dataset, as differential splicing has been shown to be a key pathogenic mechanism in tissues from a number of neurodegenerative diseases, including HD brain (27). However, analysis of individual exon, intron and transcript levels showed no significant differences in either the unstimulated or stimulated datasets (FDR < 0.05; Datasets S4–S9). This suggests that, in contrast to other tissues, differential splicing is not a major factor in mediating HD innate immune dysfunction.

### Proinflammatory functional gene sets are enriched in resting HD monocytes

**Figure 2.**
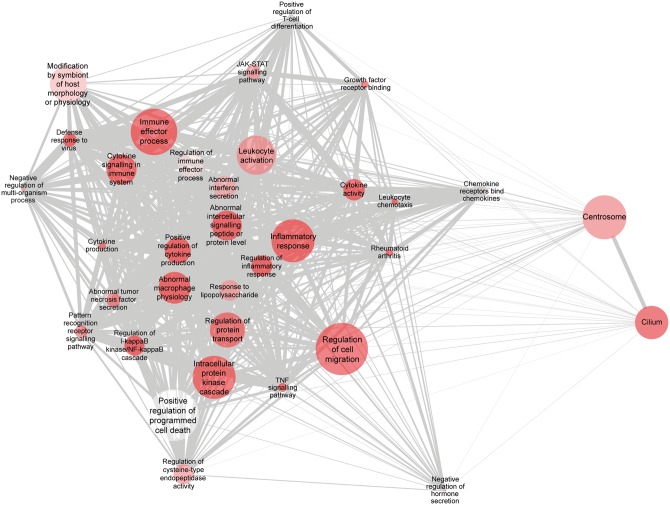
Functional gene sets associated with innate immunity and inflammation are enriched in resting HD monocytes. GSEA identified biologically relevant gene sets that are enriched in resting HD monocytes. A network diagram of significant biological themes among the upregulated genes is shown, indicating number of genes (node size), statistical significance (darkest shading = lowest *P*-value) and gene content similarity via the Jaccard coefficient (edge thickness). Nodes with similar gene content cluster more closely due to an edge-weighted layout (modified for readability). An FDR cut-off of < 0.05 was used to determine gene set inclusion in the diagram, before the Jaccard coefficient was used to filter out gene sets with similar gene content. The diagram was rendered in Cytoscape 3.3.0.

We next sought to assign biological relevance to the observed expression changes by carrying out a comprehensive Gene Set Enrichment Analysis (GSEA) of the unstimulated and stimulated datasets. GSEA allows for the unbiased identification of clusters of genes showing evidence of differential expression, without relying on the genes being individually significant after correction for multiple testing; this enables relevant biological trends to be uncovered. Over 14 000 functional gene sets drawn from multiple sources (see Materials and Methods section) were tested for enrichment; upregulated and downregulated genes were evaluated separately. Analysis of the unstimulated dataset revealed a total of 85 enriched gene sets (FDR < 0.05) amongst the upregulated genes in resting HD monocytes (Fig. 2; Dataset S10). Functional gene sets relating to innate immunity, the inflammatory response and cytokine production were heavily represented, while gene sets relating to the NFκB and JAK/STAT intracellular signalling cascades were also found to be significantly enriched. In contrast, amongst the downregulated genes only six gene sets relating to vacuole, lysosome and catabolic function were found to be significantly enriched (FDR < 0.05). Analysis of the stimulated dataset did not reveal any enriched gene sets among the upregulated genes (FDR < 0.05); this is consistent with the loss of significance for individual genes described earlier. However, 83 functional gene sets were found to be significantly enriched among the downregulated genes (Supplementary Material, Figure S1; Dataset S11); pathways relating to translation, protein localization, cholesterol homeostasis and cellular components including the cellular membrane, mitochondria and lysosome were among the most affected. Details of the genes contained in each significant gene set are included in Dataset S12.

### Specific upstream intracellular signalling pathways appear to mediate basal differences in HD monocyte gene expression

To increase our understanding of the mechanisms underlying differential gene expression in resting HD monocytes, we performed upstream regulator analysis on the unstimulated dataset using Ingenuity pathway analysis (IPA) software. IPA compares gene expression in a dataset with lists of genes that are known to be regulated by specific upstream signalling molecules, in order to infer whether a particular regulatory molecule is likely to be activated or inhibited. It is important to note that due to overlapping gene sets there is redundancy in the analysis, and that multiple points along a pathway may be suggested as upstream regulators, even if only one is driving the change. This effect is quantified by the activation z-score statistic, which increases or decreases depending on the inferred activation/inhibition state of a particular upstream regulator.

**Figure 3.**
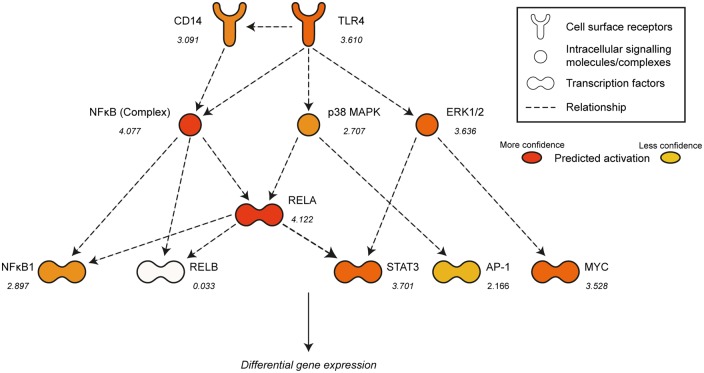
Specific intracellular signalling pathways are predicted to be abnormally activated in resting HD monocytes. IPA analysis of upstream regulators of gene transcription identified numerous signalling pathway components downstream of the TLR4 receptor which are predicted to be abnormally activated in resting HD monocytes. Upstream regulators with significantly inferred activation are shown in orange (darker colour = higher activation z-score). The activation z-score for each component is also shown (>2 or < −2 is considered to be significant).

Significance in IPA is typically attributed to upstream regulators that have an overlap *p*-value of < 0.01 and an activation z-score of > 2 or <−2; 155 upstream regulators fulfilled these criteria in our unstimulated dataset (Table 2; Dataset S13). Of these, 125 were predicted to be significantly activated, while thirty were predicted to be significantly inhibited. Due to the redundancy associated with IPA it is unlikely that this many are actually affected, so we decided to narrow our focus to the subset of upstream regulators with the highest activation z-scores, and therefore the strongest evidence for their mediating transcriptional dysregulation. Crucially, a large number of molecules associated with intracellular signalling pathways downstream of the TLR4 receptor were represented in this group (Fig. 3). Consistent with previous data showing NFκB dysregulation in HD myeloid cells, both RELA and the NFκB complex were featured in the top ten most significant results ranked by activation z-score. Other notable potential regulators included NFκB1, the ERK and p38 MAPKs, in addition to the transcription factor STAT3. These data suggests that the transcriptional changes observed in the RNA-Seq dataset are related to the abnormal activation of specific upstream signalling molecules responsible for driving gene expression in resting HD myeloid cells.

**Table 2.** IPA of upstream transcriptional regulators in resting HD monocytes

Upstream regulator	Activation z-score	*P*-value of overlap	Function
TNF	5.764	2.18E-13	Proinflammatory cytokine
IL1B	5.754	8.08E-14	Proinflammatory cytokine
MyD88	4.761	2.92E-12	Cytoplasmic adaptor protein for the TLR family
IFN-γ	4.635	3.09E-07	Cytokine involved in mediating the innate immune response
IL1A	4.536	1.69E-11	Proinflammatory cytokine
TLR3	4.323	1.99E-09	Cell surface receptor involved in the activation of the innate immune response
RELA	4.122	5.86E-08	Transcription factor; one of five NFκB family members
NFκB (complex)	4.077	2.63E-08	A family of five transcription factors and their regulators that mediate the inflammatory response
TLR9	3.773	4.33E-07	Cell surface receptor involved in the activation of the innate immune response
TLR2	3.770	3.94E-09	Cell surface receptor involved in the activation of the innate immune response
STAT3	3.701	2.34E-07	Transcription factor
IL6	3.654	7.39E-06	Proinflammatory cytokine
TICAM1/TRIF	3.651	2.59E-08	TLR cytoplasmic adaptor protein; mediates an alternative pathway to MyD88
ERK1/2	3.636	5.33E-06	Protein kinase intracellular signalling molecules
TLR4	3.610	7.68E-12	Cell surface receptor involved in the activation of the innate immune response

IPA was used to identify transcriptional regulators with significant target gene overlap with changes in the resting HD monocyte transcriptome. A P-value cut-off of < 0.01 was used to determine which genes were included in the analysis. The upstream regulators were ranked by activation z-score and the 15 most significant were included in the table.

### NFĸB but not ERK or p38 MAPK signalling is abnormally activated in resting HD myeloid cells

**Figure 4.**
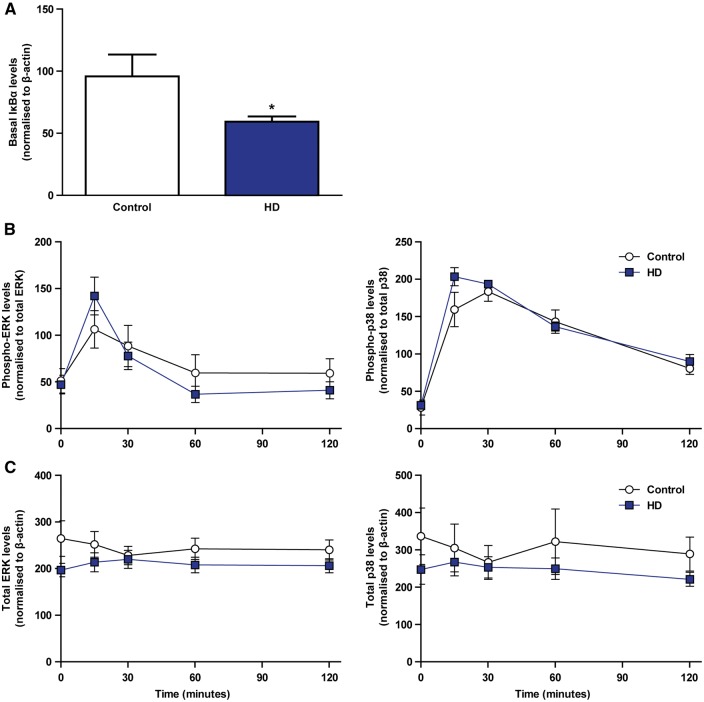
NFĸB but not ERK or p38 MAPK signalling is abnormally activated in resting HD myeloid cells. Monocyte-derived macrophages were isolated from HD and control peripheral blood samples and pulsed with LPS and IFN-γ for 15, 30, 60 and 120 min, or left unstimulated. Western blotting was then carried out on the unstimulated samples to quantify the basal levels of IĸBα, while additional Western blotting was carried out on all samples to quantify the levels of total and active, phosphorylated ERK and p38 MAPK. **(A)** Resting HD myeloid cells express significantly reduced IĸBα protein compared with control (control *n* = 7, HD *n* = 9). However, no significant differences were seen in **(B)** the ratio of phosphorylated to total enzyme, or **(C)** the ratio of total enzyme to β-actin for either ERK or p38 at any of the time points that were studied (*n* = 10). Error bars represent ± SEM, *P < 0.05.

Previously we demonstrated that HD myeloid cells exhibit an exaggerated NFκB signalling response to LPS, both in the magnitude of the initial activation and the prolonged time taken for activation to return to baseline (10). However, it is unclear whether NFĸB dysfunction also extends to the cells in their basal, resting state. To follow up the upstream regulator analysis, we next investigated whether the NFĸB pathway is abnormally activated in unstimulated HD myeloid cells. Monocyte-derived macrophages were obtained from manifest HD and control subjects, before Western blotting was carried out to quantify the expression of IĸBα. IĸBα is a cytoplasmic inhibitor of the NFĸB family of transcription factors; lower IĸBα levels are used to demonstrate increased NFĸB activity, as reduced inhibition will lead to increased translocation of NFĸB to the nucleus. Consistent with the upstream regulator analysis, we found that resting HD myeloid cells express significantly less IĸBα than control cells (Fig. 4). This demonstrates that the abnormal NFĸB activity previously described in stimulated HD myeloid cells also exists basally.

However, the contribution of alternative signalling pathways to HD myeloid cell dysfunction is yet to be fully characterized. The ERK and p38 MAPKs were selected for investigation as they both play a major role in signalling downstream of the LPS receptor TLR4 (24), and were identified by IPA as potentially significant transcriptional regulators in resting HD monocytes. ERK and p38 are both activated by phosphorylation; quantification of phosphorylated relative to total protein therefore allows their activation states to be investigated. Monocyte-derived macrophages were obtained from manifest HD and control subjects, before cells from each subject were pulsed with LPS and IFN-γ for 15, 30, 60 and 120 min, or left unstimulated. Western blotting to quantify the expression of the total and active, phosphorylated forms of both ERK and p38 showed no significant differences in activation either basally or at any of the time points following LPS stimulation. Furthermore, no significant differences were seen in the expression of total ERK or p38 compared with the reference protein β-actin. This suggests that, in contrast to NFκB, HD myeloid cells do not exhibit abnormally activated ERK or p38 signalling, and that these pathways are unlikely to drive the transcriptional changes seen in the RNA-Seq dataset.

## Discussion

Previous studies of peripheral HD myeloid cells have primarily demonstrated phenotypic dysfunction in the stimulated state (10–12). In contrast, very little is known about the extent to which these cells also display abnormalities in their basal, resting state. Here, use of RNA-Seq demonstrates that the basal transcriptome of resting HD monocytes is significantly altered, suggesting that mHTT expression has a state-independent effect occurring via a mechanism that is not restricted to the dysregulation of intracellular signalling pathways following stimulation. This has previously been shown in a wide range of tissues including brain, skeletal muscle and whole blood (22), reinforcing the observation that mHTT consistently affects the basal transcriptome in both patients and animal models of HD. Crucially, HD monocytes expressed basally elevated levels of numerous proinflammatory cytokine gene transcripts, for the first time showing increased resting cytokine expression at either the mRNA or protein level. HD patients have elevated levels of cytokines and chemokines in their peripheral blood, with increased IL-6 being detectable up to 16 years before the predicted onset of motor symptoms (6,7); the upregulated *IL6* expression seen in our dataset strongly suggests that peripheral myeloid cells are a key source. The existence of proinflammatory transcriptional changes in the absence of stimulation was confirmed by GSEA, whereby significant enrichment of numerous functional gene sets relating to innate immunity, inflammation and cytokine production was found in resting HD monocytes. This is important as it allows us to assign wider biological relevance to the expression changes seen in our dataset, while avoiding any investigator bias that could occur from looking at specific genes in isolation. Consistent with a previous RNA-Seq study on post-mortem brain, we also found that the majority of the transcriptional changes in HD monocytes involved upregulation (30).

Mutant HTT expression has been shown to promote cell-autonomous dysfunction in murine microglial cultures and human monocyte models (10,14). Furthermore, in a murine HD microglial line it has been suggested to prime resting cells, whereby the enrichment of proinflammatory molecules in the resting state leads to an exaggerated response once a stimulus is encountered. This is thought to occur via increased expression of proinflammatory transcription factors including PU.1 (14). The transcriptional dysregulation we have demonstrated in resting HD monocytes suggests that mHTT expression has a similar priming effect on primary human myeloid cells. This is a possible explanation for the increased cytokine release seen after LPS stimulation (10), and may have significant relevance to the pathobiology of HD in humans. Although HD patients have elevated levels of circulating proinflammatory cytokines (6), there is no evidence that they have an increased incidence of infectious or inflammatory events. In addition, the phenotypic improvement produced by modulation of the immune system in animal models of HD is not dependent on ameliorating artificial inflammatory stimuli (16–18). This suggests that the proinflammatory peripheral changes observed in HD *in vivo* are not dependent on, or primarily caused by, excessive cytokine release from repeatedly stimulated cells. Instead, a chronic increase in the basal release of proinflammatory mediators allows these observations to be reconciled independently of stimulation. Furthermore, a number of the cytokines known to be upregulated in HD peripheral blood are pre-synthesized in the resting state. TNFα is synthesized as a membrane bound precursor that is cleaved in response to stimulation (28), while IL-8 undergoes vesicular storage prior to release (29). Priming of HD myeloid cells by mHTT could therefore cause increased cytokine storage ready for release following stimulation, or indeed increased basal release in the absence of stimulation. Indeed, their increase in basal cytokine expression suggests that primed myeloid cells are likely to have the latter effect. It is likely that this phenomenon is also present in pre-manifest HD patients, as immune changes including increased cytokine release are detectable long before the onset of motor symptoms (6), and the short life span of peripheral blood cells precludes the possibility of a chronic effect built up over many years. This is supported by the clear existence of intrinsic mHTT-related dysfunction in HD myeloid cells, as it has been shown that peripheral immune changes occur independently of neurodegeneration, and therefore are unlikely to be solely linked to advancing central disease. The fact that numerous studies have demonstrated a disease-modifying role for the peripheral immune system raises the possibility that HD myeloid cells may even play a role in modifying disease onset (16–19), but this will require further investigation in mouse models of the disease.

Surprisingly, the transcriptome of stimulated HD monocytes was comparatively unaffected in terms of differential gene expression. Comparison of the unstimulated and stimulated log2 fold changes revealed that, while the directions of effect were largely consistent, the relative size of the majority of the fold changes was reduced under stimulated conditions to the point that they were no longer significant. This may be due to the extremely strong activating response provided by LPS, resulting in a plateau in transcriptional activity as the cellular machinery reaches the limits of its mRNA synthesis capability. This is likely to result in the masking of biological differences between HD and control monocytes, as the proinflammatory phenotype displayed by resting HD monocytes is ablated by the stronger inflammatory stimulus provided by LPS. Regardless, the lack of differentially expressed genes in the stimulated samples suggests that the proinflammatory transcriptional profile displayed by resting HD monocytes is likely to be even more crucial in mediating their hyper-reactive response to LPS. Interestingly, a number of gene sets were found to be enriched among the downregulated genes in LPS-stimulated HD monocytes. The enrichment for downregulation in numerous gene sets relating to protein localization and targeting is consistent with known cytoskeletal defects in HD (30), while further enriched gene sets were associated with organelles which are known to be affected by mHTT, including the mitochondria and lysosome (31,32). Although this analysis demonstrates that the transcriptome of stimulated HD monocytes is affected by mHTT, the relative lack of significant individual gene expression changes suggests this gene set enrichment may not have the same biological significance as that seen in resting HD myeloid cells.

Although this dataset suggests that mHTT expression has a priming effect on the transcriptome of HD monocytes, understanding the underlying mechanism requires investigation of the intracellular signalling pathways that are driving the transcriptional changes. Previous work has demonstrated that mHTT interacts directly with IKK, a key component of the NFκB pathway (23). This results in reduced binding of NFκB by its cytoplasmic inhibitor IκB, leading to increased translocation of NFκB to the nucleus and an increase in the expression of proinflammatory genes by HD myeloid cells (10). However, it was not previously known whether this dysfunction also exists in the resting state. Upstream regulator analysis using IPA inferred highly significant basal activation of RELA, NFκB1 and the NFĸB complex in resting HD monocytes. Furthermore, functional validation of IPA using Western blotting demonstrated that resting HD myeloid cells express significantly reduced levels of the NFĸB inhibitor IĸBα, showing that basal NFĸB signalling is also affected in these cells. These data provide strong support for the priming hypothesis, as the abnormal resting NFĸB activation by mHTT is likely to play a key role in mediating the proinflammatory transcriptional changes seen in the RNA-Seq dataset.

As there is a considerable degree of redundancy in IPA it is likely that the activation of a number of other upstream regulators will have been inferred as a result of their target gene overlap with NFκB. Indeed, 13 of the top 15 inferred upstream regulators ranked by activation z-score are known to be involved in NFκB signalling (whether as an activating cytokine, cell surface receptor or downstream transcription factor). This conclusion is further supported by the GSEA analysis, which identified an enrichment of genes relating to the NFĸB cascade in resting HD monocytes. Other notable IPA hits included the ERK and p38 MAPKs, and the transcription factor STAT3. However, a previous study found no evidence of increased JAK/STAT activation in HD monocytes (33), so it is likely that STAT3 activation was inferred as a result of its considerable target gene overlap with NFκB (34). Furthermore, we carried out a time-course experiment to examine the activation of the ERK and p38 MAPKs in HD myeloid cells. Although we have previously demonstrated that HD myeloid cells exhibit an exaggerated and prolonged NFκB activation response to LPS (10), no such effect was seen for ERK or p38 either basally or in response to LPS. Although this by no means rules out additional pathogenic interactions of mHTT with the transcriptional machinery, taken together these data further suggest that the transcriptional changes observed in our dataset are likely to be due in large part to resting dysregulation of the NFκB pathway.

However, it is important to note that not all transcriptional changes observed in HD are pathological, as some may be compensatory or even irrelevant (22). In addition, mHTT is known to cause transcriptional dysregulation by a variety of mechanisms, including but not limited to sequestration of transcription factors, chromatin remodelling and direct binding to DNA (22). Therefore, it should not be expected that all of the expression changes in our dataset will be explained by alterations in intracellular signalling pathways, as some will presumably be the result of the myriad other effects of mHTT on the cellular transcriptome. Despite this, upstream regulator analysis remains an important tool for studying the biology of HD immune cells, particularly when combined with established functional data.

Furthermore, this study reinforces the conclusion that the pre-eminent pathogenic mechanisms of mHTT may vary depending on cell type. Increased expression of the transcription factor PU.1 was found to mediate proinflammatory transcriptional activation in a mouse microglial model of HD (14); however, neither that study not our current study revealed any increase in PU.1 expression in HD patient monocytes. Furthermore, activation of PU.1 in our dataset was not inferred by IPA. Despite this, we have shown that resting HD monocytes have a proinflammatory transcriptional profile which may be caused by a similar mHTT priming effect to that seen in the murine HD microglial cell line. Although it has been suggested that the separate developmental origins of microglia are responsible for the prominent role of PU.1 (14), we also suggest that the relative importance of intracellular signalling pathways in different cell types helps determine their varying contributions to HD pathogenesis. The mechanism of mHTT interaction with each signalling pathway seems unlikely to be drastically altered between cell types; this can be seen in the fact that NFκB binding motifs were also found to be enriched in the HD microglial line (14). However, as the PU.1 and NFκB pathways are vital for microglial and monocyte/macrophage cell function, respectively, it is not surprising that their dysregulation by mHTT will cause them to have a central role in mediating mHTT-related phenotypic dysfunction in these cell types. Conversely, cells where these pathways have less important roles seem unlikely to experience comparable dysfunction as a result of the same mechanism, if indeed they are dysfunctional at all. Indeed, it has been shown that the intrinsic function of HD T lymphocytes is not affected by mHTT expression (13); it is possible that this is due to the reduced importance of mHTT-affected signalling pathways in mediating T lymphocyte function.

Finally, we demonstrate the importance of studying specific cell populations using modern sequencing techniques and large cohorts in order to accurately characterize HD-associated transcriptional changes. Previous transcriptional studies of HD peripheral immune cells have investigated heterogeneous cell populations with varying transcriptional profiles, and it has often not been possible to validate differentially expressed genes between studies (21,25,26). The advent of high-throughput sequencing has provided dramatically improved coverage of the entire transcriptome when compared with traditional microarrays, while the study of a single cell population allows the noise associated with studying multiple cell populations with varying transcriptional profiles to be eliminated. Although the practical considerations associated with recruiting large patient cohorts are far from trivial, the importance of studying human tissues is demonstrated by the fact that genomic studies in mice have been found to poorly mimic human inflammatory conditions (35). The study of individual cell populations is also particularly important given that a consistent transcriptional signature has not yet been established between different HD tissues (22), and observations made in one cell type may not hold true when studying another.

This study therefore provides the first evidence of significant basal proinflammatory changes in resting peripheral HD myeloid cells, with increased expression of proinflammatory cytokine mRNA in addition to the enrichment of functional gene sets associated with innate immunity, inflammation and cytokine production. This suggests that mHTT primes HD myeloid cells, resulting in an exaggerated proinflammatory response once a stimulus is encountered. We show that previously described NFĸB dysfunction also extends to resting HD myeloid cells, and that abnormal activation of this pathway is likely to play a key role in mediating their basal proinflammatory transcriptional profile. However, the ERK and p38 MAPK pathways do not appear to play a comparable role. The existence of basal dysfunction further supports the possibility that modulation of the innate immune system may be used as a therapeutic strategy to modify HD progression, as the beneficial effects of immunomodulatory drugs are likely to extend beyond ameliorating the consequences of time-limited infectious or inflammatory events. Finally, this study reinforces the importance of systemic immunity in the wider study of neurodegeneration, as the existence of *ex vivo* myeloid cell abnormalities shows that peripheral tissues are in fact intrinsically abnormal, and are not merely responding to central pathology.

## Materials and Methods

### Collection and classification of human samples

All experiments were performed in accordance with the Declaration of Helsinki and approved by the University College London (UCL)/UCL Hospitals Joint Research Ethics Committee. Peripheral blood samples were donated by genetically diagnosed HD patients and control subjects, and all subjects provided informed written consent. HD subjects were classified by their total functional capacity (TFC) score (36); only manifest patients with early or moderate stage disease (TFC 13-3) were included. The HD and control groups were age-matched (Supplementary Material, Table S1), and all subjects who donated samples for gene expression analysis were non-smokers to control for the effects of smoking on the monocyte transcriptome (37). Subjects with inflammatory or infectious conditions, and those on immunomodulatory medications, were likewise excluded from the study.

### Isolation of human monocytes

Human peripheral blood mononuclear cells (PBMCs) were isolated by density centrifugation. Peripheral blood samples were layered on Histopaque-1077 (Sigma-Aldrich) and centrifuged at 400 ×g for 30 min. CD14^+ ^monocytes were isolated by resuspending the PBMCs in 10 µl anti-human CD14 beads (Miltenyi Biotec) and 90 µl MACS buffer (PBS with 1% BSA and 2 mM EDTA) per 10^7^ cells and incubating for 15 min at 4 °C. Magnetic cell sorting was carried out by placing MACS columns (Miltenyi Biotec) in a magnetic field and adding each cell suspension to an individual column. The columns were washed with MACS buffer before magnetically labelled CD14^+ ^monocytes were eluted by removing the columns from the magnetic field and plunging MACS buffer through the column. The sorted cell populations contained an average of 85–95% CD14^+ ^cells.

### Cell culture and stimulation

Cells were cultured in RPMI 1640 culture medium supplemented with 10% FBS, 2 mM L-glutamine, 50 U/ml penicillin and 50 µg/ml streptomycin (Thermo Fisher Scientific). Monocytes were allowed to rest for 16 h post-seeding before experimental use. Monocyte-derived macrophages were obtained by supplementing the culture medium with 20 ng/ml granulocyte macrophage-colony stimulating factor (R&D Systems) for 6 days to induce differentiation prior to experimental use. Monocyte cultures were stimulated with 2 µg/ml LPS (Sigma-Aldrich) and 10 ng/ml IFN-γ (R&D Systems), while macrophage cultures were stimulated with 1 µg/ml LPS and 10 ng/ml IFN-γ. Monocyte stimulation for RNA-Seq analysis was carried out for 4 h, as this has previously been shown to be the peak time point for TLR4-mediated gene activation following LPS stimulation (38,39).

### RNA preparation and sequencing

RNA was extracted using the RNeasy Mini Kit (QIAGEN), including the optional DNase step to prevent genomic DNA contamination. RNA integrity was assessed using RNA 6000 Nano Chips on a 2100 Bioanalyser (Agilent). Only samples with non-degraded RNA (RIN ≥ 7) were used for sequencing. Sequencing of RNA samples was performed by deCODE Genetics, Iceland. Preparation of indexed cDNA sequencing libraries was carried out using the TruSeq poly-A mRNA method (Illumina). Briefly, poly-A mRNA transcripts were captured from total RNA using poly-T beads, before cDNA was generated using random hexamer priming. Paired-end sequencing (2 × 100 cycles) of indexed cDNA libraries was then carried out on a HiSeq 2500 machine (Illumina), generating at least 50 million reads (101 base pairs) per sample. Sequencing was performed using v4 SBS and Cluster Kits (Illumina). One sample failed the library generation step and was excluded from the study. After sequencing the indexed samples were demultiplexed before generation of FASTQ files for analysis. Raw data files are available from the European Nucleotide Archive (http://www.ebi.ac.uk/ena) under the study accession number PRJEB12995.

### Data processing and expression analysis

Data quality control was performed using the RNA-SeQC package (40). Metrics including rRNA rate, mapping rate, concordance mapping rate and uniqueness rate were determined to confirm that they were within acceptable parameters. One sample failed quality control and was removed from further analysis. After quality control the remaining samples were aligned using TopHat2 (41), before the counts were summarized using HTSeq (keeping any read duplicates). Differential expression analysis was then performed using the R package DESeq2 (42). Outlier counts were removed using a Cooks distance cut-off of 5 in DESeq2 packages and gender was used as a covariate in the analysis. Differential splicing analysis was carried out on the aligned data using the R package Ballgown (43).

### Gene set enrichment analysis

Enrichment of differential expression among gene sets corresponding to biological hypotheses (pathways) was tested using the GSEA method (44). Rather than defining a list of significant genes, GSEA ranks all genes in order of their differential expression statistic, and tests whether the genes in a particular gene set have a higher rank overall than would be expected by chance. The analysis is weighted by the differential expression statistic, thus giving more weight to more significant genes. Significance of enrichment was obtained by randomly permuting gene-wide association statistics among genes. One-sided *p*-values were calculated separately for differential upregulation and downregulation of expression in HD, and these were then converted into the corresponding chi-square statistic for use in the GSEA analysis. To avoid making *a priori* assumptions, we chose to use a large pathway set comprising Gene Ontology (GO) (45), Kyoto Encyclopedia of Genes and Genomes (46), PANTHER v8.1 (47), Mouse Genome Informatics (MGI) (48), Reactome (49), Biocarta and NCIpathway interaction database (50) pathways. This resulted in a total of 14 243 functional gene sets, many with overlapping members, containing between 3 and 500 genes. To correct for multiple testing, we converted the GSEA *P*-values into *q*-values (51), which can be interpreted as the minimum false discovery rate (FDR) at which that *q*-value would be counted as significant.

### Upstream regulator analysis

Upstream regulator analysis was carried out using QIAGEN’s IPA. IPA identifies potential upstream regulators of transcriptional change using the *P*-value of overlap and activation z-score statistics. The *P*-value of overlap uses Fisher’s exact test to determine whether there is statistically significant overlap between gene expression changes in a dataset and the genes which are affected by an upstream regulator; significance is attributed to molecules with a *P*-value of < 0.01. This does not take the direction of expression changes into account, so the activation z-score is used to predict whether potential upstream regulators are activated or inhibited in the dataset. A z-score of > 2 or < −2 is generally considered to be significant. Further information on how the activation z-score is calculated is available on the IPA website (http://www.ingenuity.com/, Last accessed March 29, 2016). A *P*-value cut-off of 0.01 was used to determine which genes were included in the analysis.

### Western blotting

Cells were lysed in RIPA buffer (25 mM Tris-HCl (pH 7.6), 150 mM NaCl, 1% NP-40, 1% sodium deoxycholate, 0.1% SDS and 1X complete protease inhibitor cocktail (Roche)), mixed with Laemmli buffer and denatured at 95 °C for 10 min. Lysates were run on 12% Tris-Glycine gels (Invitrogen) at 120 V, before transfer to 0.2 µm nitrocellulose membranes at 35 V for 2 h. Membranes were blocked in 1:1 PBS/Odyssey Blocking Buffer (LI-COR) for 1 h before incubation with primary antibody diluted in 1:1 PBS/Odyssey Blocking Buffer at 4 °C overnight. Primary antibodies used were to IĸBα (Santa Cruz Biotech; 1:500), p38, phospho-p38, ERK (p42/44), phospho-ERK (p42/44) (all Cell Signalling Technology; 1:1000) and β-actin (Abcam; 1:10000). After washing with PBS the membranes were incubated with secondary antibody diluted in 1:1 PBS/Odyssey Blocking Buffer for 1 h. Secondary antibodies used were IRDye 680RD Goat Anti-Mouse IgG and IRDye 800CW Goat Anti-Rabbit IgG (LI-COR; 1:5000). After washing the signal was visualized using an Odyssey Infrared Imager (LI-COR) and quantified using TL100 software (TotalLab).

### Statistical analysis

Statistical analysis of differential expression, splicing, GSEA and IPA data was carried out using the software packages outlined above. IĸBα Western blotting was analysed using unpaired two-tailed Student’s *t*-tests, while ERK and p38 MAPK Western blotting experiments were analysed by two-way ANOVAs with Bonferroni post-tests. All error bars represent SEM.

## Supplementary Material

Supplementary Material is available at *HMG* online.

Supplementary Data

## References

[1] The Huntington’s Disease Collaborative Research Group. (1993) A novel gene containing a trinucleotide repeat that is expanded and unstable on Huntington’s disease chromosomes. Cell, 72, 971–983. 8458085 10.1016/0092-8674(93)90585-e

[2] Ross C.A. Tabrizi S.J. (2011) Huntington's disease: from molecular pathogenesis to clinical treatment. Lancet Neurol., 10, 83–98. 21163446 10.1016/S1474-4422(10)70245-3

[3] Li S.H. Schilling G. Young W.S. Li X.J. Margolis R.L. Stine O.C. Wagster M.V. Abbott M.H. Franz M.L. Ranen N.G. (1993) Huntington's disease gene (IT15) is widely expressed in human and rat tissues. Neuron, 11, 985–993. 8240819 10.1016/0896-6273(93)90127-d

[4] van der Burg J.M. Björkqvist M. Brundin P. (2009) Beyond the brain: widespread pathology in Huntington's disease. Lancet Neurol., 8, 765–774. 19608102 10.1016/S1474-4422(09)70178-4

[5] Tai Y.F. Pavese N. Gerhard A. Tabrizi S.J. Barker R.A. Brooks D.J. Piccini P. (2007) Microglial activation in presymptomatic Huntington's disease gene carriers. Brain, 130, 1759–1766. 17400599 10.1093/brain/awm044

[6] Björkqvist M. Wild E.J. Thiele J. Silvestroni A. Andre R. Lahiri N. Raibon E. Lee R.V. Benn C.L. Soulet D, (2008) A novel pathogenic pathway of immune activation detectable before clinical onset in Huntington's disease. J. Exp. Med., 205, 1869–1877. 18625748 10.1084/jem.20080178 PMC2525598

[7] Wild E. Magnusson A. Lahiri N. Krus U. Orth M. Tabrizi S.J. Björkqvist M. (2011) Abnormal peripheral chemokine profile in Huntington's disease. PLoS Curr., 3, RRN1231. 21826115 10.1371/currents.RRN1231 PMC3082446

[8] Politis M. Lahiri N. Niccolini F. Su P. Wu K. Giannetti P. Scahill R.I. Turkheimer F.E. Tabrizi S.J. Piccini P. (2015) Increased central microglial activation associated with peripheral cytokine levels in premanifest Huntington's disease gene carriers. Neurobiol. Dis., 83, 115–121. 26297319 10.1016/j.nbd.2015.08.011

[9] Ellrichmann G. Reick C. Saft C. Linker R.A. (2013) The role of the immune system in Huntington's disease. Clin. Dev. Immunol., 2013, 541259. 23956761 10.1155/2013/541259 PMC3727178

[10] Träger U. Andre R. Lahiri N. Magnusson-Lind A. Weiss A. Grueninger S. McKinnon C. Sirinathsinghji E. Kahlon S. Pfister E.L (2014) HTT-lowering reverses Huntington's disease immune dysfunction caused by NFκB pathway dysregulation. Brain, 137, 819–833. 24459107 10.1093/brain/awt355 PMC3983408

[11] Kwan W. Träger U. Davalos D. Chou A. Bouchard J. Andre R. Miller A. Weiss A. Giorgini F. Cheah C (2012) Mutant huntingtin impairs immune cell migration in Huntington disease. J. Clin. Invest., 122, 4737–4747. 23160193 10.1172/JCI64484 PMC3533551

[12] Träger U. Andre R. Magnusson-Lind A. Miller J.R. Connolly C. Weiss A. Grueninger S. Silajdžić E. Smith D.L. Leavitt B.R (2015) Characterisation of immune cell function in fragment and full-length Huntington’s disease mouse models. Neurobiol. Dis., 73, 388–398. 25447230 10.1016/j.nbd.2014.10.012 PMC4262574

[13] Miller J.R. Träger U. Andre R. Tabrizi S.J. (2015) Mutant Huntingtin Does Not Affect the Intrinsic Phenotype of Human Huntington's Disease T Lymphocytes. PLoS One, 10, e0141793. 26529236 10.1371/journal.pone.0141793 PMC4631523

[14] Crotti A. Benner C. Kerman B.E. Gosselin D. Lagier-Tourenne C. Zuccato C. Cattaneo E. Gage F.H. Cleveland D.W. Glass C.K. (2014) Mutant Huntingtin promotes autonomous microglia activation via myeloid lineage-determining factors. Nat. Neurosci., 17, 513–521. 24584051 10.1038/nn.3668 PMC4113004

[15] Weiss A. Träger U. Wild E.J. Grueninger S. Farmer R. Landles C. Scahill R.I. Lahiri N. Haider S. Macdonald D (2012) Mutant huntingtin fragmentation in immune cells tracks Huntington's disease progression. J. Clin. Invest., 122, 3731–3736. 22996692 10.1172/JCI64565 PMC3461928

[16] Bouchard J. Truong J. Bouchard K. Dunkelberger D. Desrayaud S. Moussaoui S. Tabrizi S.J. Stella N. Muchowski P.J. (2012) Cannabinoid receptor 2 signaling in peripheral immune cells modulates disease onset and severity in mouse models of huntington’s disease. J. Neurosci., 32, 18259–18268. 23238740 10.1523/JNEUROSCI.4008-12.2012 PMC3753072

[17] Hsiao H.Y. Chiu F.L. Chen C.M. Wu Y.R. Chen H.M. Chen Y.C. Kuo H.C. Chern Y. (2014) Inhibition of soluble tumor necrosis factor is therapeutic in Huntington's disease. Hum. Mol. Genet., 23, 4328–4344. 24698979 10.1093/hmg/ddu151

[18] Zwilling D. Huang S.Y. Sathyasaikumar K.V. Notarangelo F.M. Guidetti P. Wu H.Q. Lee J. Truong J. Andrews-Zwilling Y. Hsieh E.W (2011) Kynurenine 3-monooxygenase inhibition in blood ameliorates neurodegeneration. Cell, 145, 863–874. 21640374 10.1016/j.cell.2011.05.020 PMC3118409

[19] Kwan W. Magnusson A. Chou A. Adame A. Carson M.J. Kohsaka S. Masliah E. Möller T. Ransohoff R. Tabrizi S.J (2012) Bone marrow transplantation confers modest benefits in mouse models of Huntington’s disease. J. Neurosci., 32, 133–142. 22219276 10.1523/JNEUROSCI.4846-11.2012 PMC3571858

[20] Hodges A. Strand A.D. Aragaki A.K. Kuhn A. Sengstag T. Hughes G. Elliston L.A. Hartog C. Goldstein D.R. Thu D (2006) Regional and cellular gene expression changes in human Huntington’s disease brain. Hum. Mol. Genet., 15, 965–977. 16467349 10.1093/hmg/ddl013

[21] Borovecki F. Lovrecic L. Zhou J. Jeong H. Then F. Rosas H.D. Hersch S.M. Hogarth P. Bouzou B. Jensen R.V (2005) Genome-wide expression profiling of human blood reveals biomarkers for Huntington’sdisease. Proc. Natl. Acad. Sci. USA, 102, 11023–11028. 16043692 10.1073/pnas.0504921102 PMC1182457

[22] Seredenina T. Luthi-Carter R. (2012) What have we learned from gene expression profiles in Huntington’s disease? Neurobiol. Dis., 45, 83–98. 21820514 10.1016/j.nbd.2011.07.001

[23] Khoshnan A. Ko J. Watkin E.E. Paige L.A. Reinhart P.H. Patterson P.H. (2004) Activation of the IkappaB kinase complex and nuclear factor-kappaB contributes to mutant huntingtin neurotoxicity. J. Neurosci., 24, 7999–8008. 15371500 10.1523/JNEUROSCI.2675-04.2004 PMC6729796

[24] Akira S. Takeda K. (2004) Toll-like receptor signalling. Nat. Rev. Immunol., 4, 499–511. 15229469 10.1038/nri1391

[25] Runne H. Kuhn A. Wild E.J. Pratyaksha W. Kristiansen M. Isaacs J.D. Régulier E. Delorenzi M. Tabrizi S.J. Luthi-Carter R. (2007) Analysis of potential transcriptomic biomarkers for Huntington’s disease in peripheral blood. Proc. Natl. Acad. Sci. USA, 104, 14424–14429. 17724341 10.1073/pnas.0703652104 PMC1964868

[26] Mastrokolias A. Ariyurek Y. Goeman J.J. van Duijn E. Roos R.A. van der Mast R.C. van Ommen G.B. den Dunnen J.T. 't Hoen P.A. van Roon-Mom W.M. (2015) Huntington’s disease biomarker progression profile identified by transcriptome sequencing in peripheral blood. Eur. J. Hum. Genet., 23, 1349–1356. 25626709 10.1038/ejhg.2014.281 PMC4592077

[27] Fernández-Nogales M. Cabrera J.R. Santos-Galindo M. Hoozemans J.J. Ferrer I. Rozemuller A.J. Hernández F. Avila J. Lucas J.J. (2014) Huntington’s disease is a four-repeat tauopathy with tau nuclear rods. Nat. Med., 20, 881–885. 25038828 10.1038/nm.3617

[28] Black R.A. Rauch C.T. Kozlosky C.J. Peschon J.J. Slack J.L. Wolfson M.F. Castner B.J. Stocking K.L. Reddy P. Srinivasan S (1997) A metalloproteinase disintegrin that releases tumour-necrosis factor-alpha from cells. Nature, 385, 729–733. 9034190 10.1038/385729a0

[29] Lacy P. Stow J.L. (2011) Cytokine release from innate immune cells: association with diverse membrane trafficking pathways. Blood, 118, 9–18. 21562044 10.1182/blood-2010-08-265892

[30] Gunawardena S. Her L.S. Brusch R.G. Laymon R.A. Niesman I.R. Gordesky-Gold B. Sintasath L. Bonini N.M. Goldstein L.S. (2003) Disruption of axonal transport by loss of huntingtin or expression of pathogenic polyQ proteins in Drosophila. Neuron, 40, 25–40. 14527431 10.1016/s0896-6273(03)00594-4

[31] Damiano M. Galvan L. Déglon N. Brouillet E. (2010) Mitochondria in Huntington’s disease. Biochim. Biophys. Acta., 1802, 52–61. 19682570 10.1016/j.bbadis.2009.07.012

[32] Martin D.D. Ladha S. Ehrnhoefer D.E. Hayden M.R. (2015) Autophagy in Huntington disease and huntingtin in autophagy. Trends Neurosci., 38, 26–35. 25282404 10.1016/j.tins.2014.09.003

[33] Träger U. Magnusson A. Lahiri Swales N. Wild E. North J. Lowdell M. Björkqvist M. (2013) JAK/STAT signalling in Huntington’s disease immune cells. PLoS Curr., 5. 10.1371/currents.hd.5791c897b5c3bebeed93b1d1da0c0648 PMC3871417 24459609

[34] Hoesel B. Schmid J.A. (2013) The complexity of NF-κB signaling in inflammation and cancer. Mol. Cancer, 12, 86. 23915189 10.1186/1476-4598-12-86 PMC3750319

[35] Seok J. Warren H.S. Cuenca A.G. Mindrinos M.N. Baker H.V. Xu W. Richards D.R. McDonald-Smith G.P. Gao H. Hennessy L (2013) Genomic responses in mouse models poorly mimic human inflammatory diseases. Proc. Natl. Acad. Sci. USA, 110, 3507–3512. 23401516 10.1073/pnas.1222878110 PMC3587220

[36] Shoulson I. (1981) Huntington disease: functional capacities in patients treated with neuroleptic and antidepressant drugs. Neurology, 31, 1333–1335. 6125919 10.1212/wnl.31.10.1333

[37] Zeller T. Wild P. Szymczak S. Rotival M. Schillert A. Castagne R. Maouche S. Germain M. Lackner K. Rossmann H (2010) Genetics and beyond–the transcriptome of human monocytes and disease susceptibility. PLoS One, 5, e10693. 20502693 10.1371/journal.pone.0010693 PMC2872668

[38] Aung H.T. Schroder K. Himes S.R. Brion K. van Zuylen W. Trieu A. Suzuki H. Hayashizaki Y. Hume D.A. Sweet M.J (2006) LPS regulates proinflammatory gene expression in macrophages by altering histone deacetylase expression. Faseb J., 20, 1315–1327. 16816106 10.1096/fj.05-5360com

[39] Yamamoto M. Yamazaki S. Uematsu S. Sato S. Hemmi H. Hoshino K. Kaisho T. Kuwata H. Takeuchi O. Takeshige K (2004) Regulation of Toll/IL-1-receptor-mediated gene expression by the inducible nuclear protein IkappaBzeta. Nature, 430, 218–222. 15241416 10.1038/nature02738

[40] DeLuca D.S. Levin J.Z. Sivachenko A. Fennell T. Nazaire M.D. Williams C. Reich M. Winckler W. Getz G. (2012) RNA-SeQC: RNA-seq metrics for quality control and process optimization. Bioinformatics, 28, 1530–1532. 22539670 10.1093/bioinformatics/bts196 PMC3356847

[41] Kim D. Pertea G. Trapnell C. Pimentel H. Kelley R. Salzberg S.L. (2013) TopHat2: accurate alignment of transcriptomes in the presence of insertions, deletions and gene fusions. Genome Biol., 14, R36. 23618408 10.1186/gb-2013-14-4-r36 PMC4053844

[42] Love M.I. Huber W. Anders S. (2014) Moderated estimation of fold change and dispersion for RNA-seq data with DESeq2. Genome Biol., 15, 550. 25516281 10.1186/s13059-014-0550-8 PMC4302049

[43] Frazee A.C. Pertea G. Jaffe A.E. Langmead B. Salzberg S.L. Leek J.T. (2015) Ballgown bridges the gap between transcriptome assembly and expression analysis. Nat. Biotechnol., 33, 243–246. 25748911 10.1038/nbt.3172 PMC4792117

[44] Subramanian A. Tamayo P. Mootha V.K. Mukherjee S. Ebert B.L. Gillette M.A. Paulovich A. Pomeroy S.L. Golub T.R. Lander E.S (2005) Gene set enrichment analysis: a knowledge-based approach for interpreting genome-wide expression profiles. Proc. Natl. Acad. Sci. USA, 102, 15545–15550. 16199517 10.1073/pnas.0506580102 PMC1239896

[45] Harris M.A. Clark J. Ireland A. Lomax J. Ashburner M. Foulger R. Eilbeck K. Lewis S. Marshall B. Mungall C (2004) The Gene Ontology (GO) database and informatics resource. Nucleic Acids Res., 32, D258–D261. 14681407 10.1093/nar/gkh036 PMC308770

[46] Kanehisa M. Goto S. Sato Y. Furumichi M. Tanabe M. (2012) KEGG for integration and interpretation of large-scale molecular data sets. Nucleic Acids Res., 40, D1091–D1014. 10.1093/nar/gkr988 PMC3245020 22080510

[47] Mi H. Muruganujan A. Thomas P.D. (2013) PANTHER in 2013: modeling the evolution of gene function, and other gene attributes, in the context of phylogenetic trees. Nucleic Acids Res., 41, D377–D386. 23193289 10.1093/nar/gks1118 PMC3531194

[48] Bult C.J. Eppig J.T. Kadin J.A. Richardson J.E. Blake J.A. Group M.G.D. (2008) The Mouse Genome Database (MGD): mouse biology and model systems. Nucleic Acids Res., 36, D724–D728. 18158299 10.1093/nar/gkm961 PMC2238849

[49] Croft D. Mundo A.F. Haw R. Milacic M. Weiser J. Wu G. Caudy M. Garapati P. Gillespie M. Kamdar M.R (2014) The Reactome pathway knowledgebase. Nucleic Acids Res., 42, D472–D477. 24243840 10.1093/nar/gkt1102 PMC3965010

[50] Schaefer C.F. Anthony K. Krupa S. Buchoff J. Day M. Hannay T. Buetow K.H. (2009) PID: the Pathway Interaction Database. Nucleic Acids Res., 37, D674–D679. 18832364 10.1093/nar/gkn653 PMC2686461

[51] Storey J.D. Tibshirani R. (2003) Statistical significance for genomewide studies. Proc. Natl. Acad. Sci. USA, 100, 9440–9445. 12883005 10.1073/pnas.1530509100 PMC170937

