# Supplementary material for: RNA-Seq of Huntington’s disease patient myeloid cells reveals innate transcriptional dysregulation associated with proinflammatory pathway activation
Source: Hum Mol Genet. 2016 May 11;25(14):2893–904. doi: 10.1093/hmg/ddw142 (PMC5181590; doi:10.1093/hmg/ddw142)
Supplement: Supplementary Data [file supp_25_14_2893__index.html]

RNA-Seq of Huntington’s disease patient myeloid cells reveals innate transcriptional dysregulation associated with proinflammatory pathway activation — Supplementary Data 

# RNA-Seq of Huntington’s disease patient myeloid cells reveals innate transcriptional dysregulation associated with proinflammatory pathway activation

## Supplementary Data

files

- Supplementary Data - zip file
