# Supplementary material for: RNA-Seq of Huntington’s disease patient myeloid cells reveals innate transcriptional dysregulation associated with proinflammatory pathway activation
Source: Hum Mol Genet. 2016 May 11;25(14):2893–904. doi: 10.1093/hmg/ddw142 (PMC5181590; doi:10.1093/hmg/ddw142)
Supplement: Supplementary Data [file supp_ddw142_suppl_data.zip › Miller et al Supplementary Data.doc]

**Figure S1. Gene set enrichment analysis in stimulated HD monocytes.** GSEA was used to identify biologically relevant gene sets which are enriched in stimulated HD monocytes. A network diagram of significant biological themes among the downregulated genes is shown, indicating number of genes (node size), statistical significance (darkest shading = lowest *p*-value) and gene content similarity via the Jaccard coefficient (edge thickness). Nodes with similar gene content cluster more closely due to an edge-weighted layout (modified for readability). An FDR cut-off of < 0.05 was used to determine gene set inclusion in the diagram, before the Jaccard coefficient was used to filter out gene sets with similar gene content. The diagram was rendered in Cytoscape 3.3.0.

| **Experiment** | **Subject group** | **N** | **Age**  **(mean ± SD)** | **CAG**  **(mean ± SD)** |
| --- | --- | --- | --- | --- |
| RNA-Seq analysis of the monocyte transcriptome | Control | 33 | 48.9 ± 13.2 | - |
|  | HD | 30 | 51.3 ± 11.0 | 44.4 ± 2.8 |
| IĸBα Western blotting | Control | 7 | 52.9 ± 16.2 | - |
|  | HD | 9 | 62.1 ± 6.0 | 42.4 ± 2.6 |
| ERK and p38 Western blotting | Control | 10 | 55.3 ± 10.9 | - |
|  | HD | 10 | 61.8 ± 5.8 | 42.4 ± 2.4 |

**Table S1. Age and *HTT* CAG repeat lengths of subjects participating in the study.** The cohort used for each experiment is listed separately.

**Supplementary datasets:**

**Dataset S1. Complete differential expression analysis of unstimulated compared to LPS-stimulated monocytes.** Analysis carried out to confirm LPS-stimulation; HD and control samples were pooled. Log2 fold change shown as unstimulated relative to LPS-stimulated samples.

**Dataset S2. Complete differential expression analysis of unstimulated HD monocytes.**

**Dataset S3. Complete differential expression analysis of LPS-stimulated HD monocytes.**

**Dataset S4. Complete analysis of alternative exon splicing in unstimulated HD monocytes.**

**Dataset S5. Complete analysis of alternative exon splicing in LPS-stimulated HD monocytes.**

**Dataset S6. Complete analysis of alternative intron splicing in unstimulated HD monocytes.**

**Dataset S7. Complete analysis of alternative intron splicing in LPS-stimulated HD monocytes.**

**Dataset S8. Complete analysis of alternative transcript splicing in unstimulated HD monocytes.**

**Dataset S9. Complete analysis of alternative transcript splicing in LPS-stimulated HD monocytes.**

**Dataset S10. Complete Gene Set Enrichment Analysis of unstimulated HD monocytes.** Analyses of the upregulated (plus) and downregulated (minus) genes are shown in separate sheets.

**Dataset S11. Complete Gene Set Enrichment Analysis of stimulated HD monocytes.** Analyses of the upregulated (plus) and downregulated (minus) genes are shown in separate sheets.

**Dataset S12. Gene signature list for significant GSEA gene gets.** Genes belonging to all gene sets with FDR < 0.05 in either the unstimulated or LPS-stimulated GSEA analyses are shown. Gene signature lists for the enriched gene sets in the upregulated (plus) and downregulated (minus) analyses are shown in separate sheets.

**Dataset S13. Complete IPA® upstream regulator analysis of unstimulated HD monocytes.** A *p*-value cut-off of < 0.01 was used to determine which genes were included in the analysis.
